# Minimally Invasive Surgical Approaches and Traditional Total Hip Arthroplasty: A Meta-Analysis of Radiological and Complications Outcomes

**DOI:** 10.1371/journal.pone.0037947

**Published:** 2012-05-24

**Authors:** Baohui Yang, Haopeng Li, Xijing He, Guoyu Wang, Siyue Xu

**Affiliations:** Department of Orthopaedic Surgery, The 2nd Affiliated Hospital of Medical College, Xi'an Jiaotong University, Xi'an, Shaanxi Province, People's Republic of China; University of Michigan, United States of America

## Abstract

**Background:**

Minimally invasive total hip arthroplasty (MITHA) remains considerably controversial. Limited visibility and prosthesis malposition increase the risk of post-surgical complications compared to those of the traditional method.

**Methods:**

A meta-analysis was undertaken of all published databases up to May 2011. The studies were divided into four subgroups according to the surgical approach taken. The radiological outcomes and complications of minimally invasive surgery were compared to traditional total hip arthroplasty (TTHA) using risk ratio, mean difference, and standardized mean difference statistics.

**Results:**

In five studies involving the posterolateral approach, no significant differences were found between the MITHA groups and the TTHA groups in the acetabular cup abduction angle (p = 0.41), acetabular anteversion (p = 0.96), and femoral prosthesis position (p = 0.83). However, the femoral offset was significantly increased (WMD = 3.00; 95% CI, 0.40–5.60; p = 0.02). Additionally, there were no significant differences among the complications in both the groups (dislocations, nerve injury, infection, deep vein thrombosis, proximal femoral fracture) and revision rate (p>0.05). In three studies involving the posterior approach, there were no significant differences in radiological outcomes or all other complications between MITHA or TTHA groups (p>0.05). Three studies involved anterolateral approach, while 2 studies used the lateral approach. However, the information from imaging and complications was not adequate for statistical analysis.

**Conclusions:**

Posterior MITHA seems to be a safe surgical procedure, without the increased risk of post-operative complication rates and component malposition rates. The posterolateral approach THA may lead to increased femoral offset. The current data are not enough to reach a positive conclusion that lateral and anterolateral approaches will result in increased risks of adverse effects and complications at the prosthesis site.

## Introduction

Total hip arthroplasty (THA) is considered one of the most successful operations in orthopedic surgery for over 40 years, during which improvements in the design of implants and biological materials have resulted in positive clinical outcomes. However, for the past decade, considerable interest has been devoted to the development of minimally invasive surgical techniques [1]. Minimally invasive total hip arthroplasty (MITHA) has now become popular around the world. It is defined as the use of a 10 cm or even smaller incision to complete the total hip joint replacement [2], [3], [4].

The advantages of minimally invasive surgeries include less soft-tissue trauma (smaller skin incision and less muscle damage), reduced blood loss and fewer blood transfusion requirements. Postoperative benefits include less pain, shorter hospital stay, quicker return to function and a better cosmetic appearance [2], [4], [5].

Despite the increase in adopting MITHA, its risks and benefits still generate debates among orthopedic surgeons. Many of them believe that MITHA introduces additional risks due to the limited visibility of anatomical landmarks and vital structures [6]. Higher risks for thromboembolism, infection, neurovascular injury, femoral fracture and component malposition leading to increased prosthetic wear, are the various complications that have been documented [7]. Bradley P et al [8] retrospectively reviewed 46 revision THAs performed during a 3-year period and concluded that MITHA may be a risk factor for early revision surgery and the long-term survival therefore may be lower than that for non-minimal invasive surgery. Another drawback seems to be the learning curve, which tends to be longer for surgeons with little experience of hip prosthetic surgery. Some other randomized, case-control studies also reveal inconsistent results regarding these issues [9]–[12]. Therefore, it remains controversial whether MITHA increases post-operative complications and prosthesis malposition.

There have been several related meta-analysis studies [13], [14]. These analyses seem to have simply pooled the results together without an explicitly defined sub-group analysis of the surgical approach. Clearly, a 12 cm posterior approach would have very different risks and complications than an 8 cm direct anterior, or two incision approach. A very recent study [15] also pointed this issue. Therefore, to group all MITHA approaches together and make comparisons in a pooled analysis is unsound since differences in technique (e.g. between the mini-anterior, mini-posterior, mini-anterolateral approaches) are significant and need to be analyzed separately. The purpose of this meta-analysis was to compare the MITHA with conventional, or traditional total hip arthroplasty (TTHA) with respect to complications and post-operative results through imaging, of subgroups formed by the surgical approaches taken.

## Materials and Methods

We conducted a meta-analysis using the guidelines of the Cochrane Collaboration [16], and our findings were reported according to the Quality of Reporting of Meta-analyses statement [17].

### Types of studies included

Any randomized controlled trials comparing MITHA and TTHA for the treatment of hip disease.

### Types of Participants

Participants of the 2 treatment groups were similar demographically, and there were no statistically significant differences with respect to the variables of age, gender, body mass index (<35.0).

### Types of interventions

We focused on comparing MITHA and TTHA with a common operation approach that is, posterior, posterolateral:, anterolateral, lateral, so that there would be a consistency between the two groups.

We excluded studies in which the surgical approach was inconsistent (for example, if the minimally invasive group used the anterolateral approach, and the traditional group used the posterior approach, etc.). In addition, we also excluded studies that examined the double incision surgical approach.

### Types of outcome measures

*The primary outcomes were:*

1. Imaging outcomes (acetabular abduction angle, acetabular anteversion, femoral prosthesis position (varus or valgus), and femoral offset.
2. Postoperative complications (dislocation, iatrogenic nerve injury, infection, deep vein thrombosis, proximal femoral fracture, and revision rate).

### Search strategy

The following search terms were used: total hip arthroplasty (or THA), total hip replacement (or THR), prosthesis (or prostheses or implant), minimally invasive (or less invasive), mini-incision (or MIS or minimal incision or small incision). The literature range was defined as between February 1990 and May 2011. The databases searched included PubMed, Cochrane Central Register of Controlled Trials, Embase, and CBMdisc. A manual search was performed for relevant publications from European Federation of National Associations of Orthopaedics and Traumatology and British Hip Society.

### Data collection and analysis

Both review authors (BaoHui Yang and HaoPeng LI) assessed potentially eligible trials for inclusion, any disagreement were resolved through discussion. Titles of journals, names of authors or supporting institutions were not masked at any stage.

### Data extraction and management

Data were extracted independently by both authors using piloted forms. The data included the general characteristics of each study and the outcomes measured. General characteristics included study design, first author, year of publication, sample size, and interventions. Only the primary outcomes were measured.

### Assessment of risk of bias in included studies

To avoid inherent problems with scale validity [17], we did not use a quality scale or a checklist. We assessed the methodological quality as described by the Cochrane Reviews Handbook 5.0.2 [17], Methodological quality assessment scheme (Table S1). The studies were classified into A: low risk of bias and each of the criteria was appropriate, B: medium risk of bias and most of the criteria were appropriate, and C: high risk of bias and most of the criteria were not appropriate.

### Measures of treatment effect

For continuous data such as acetabular cup abduction angle, the mean and standard deviation were used to calculate the weighted mean difference (WMD) and 95% confidence interval (CI); for count data such as post-operative complications, the odds ratio (OR) or relative risk (RR) and 95% CI were used. Data processing was performed using Review Manager 5.0 software (the Cochrane Collaboration).

#### Assessment of heterogeneity

The heterogeneity test P values revealed by the forest plot were used to determine the heterogeneity of the included studies. I^2^ was used to estimate the size of the heterogeneity. I^2^>50% indicated considerable heterogeneity among the included studies.

### Subgroup analysis and investigation of heterogeneity

We planned separate outcome analyses according to the different surgical approaches, and if there is no heterogeneity internal each groups, data were pooled using the fixed-effect model and 95% confidence intervals. Where there was clear or significant heterogeneity, we viewed the results of the random-effects model, but in such cases opted not to pool data where the outcome measures were clearly different.

## Results

### Description of studies

**Figure 1:**
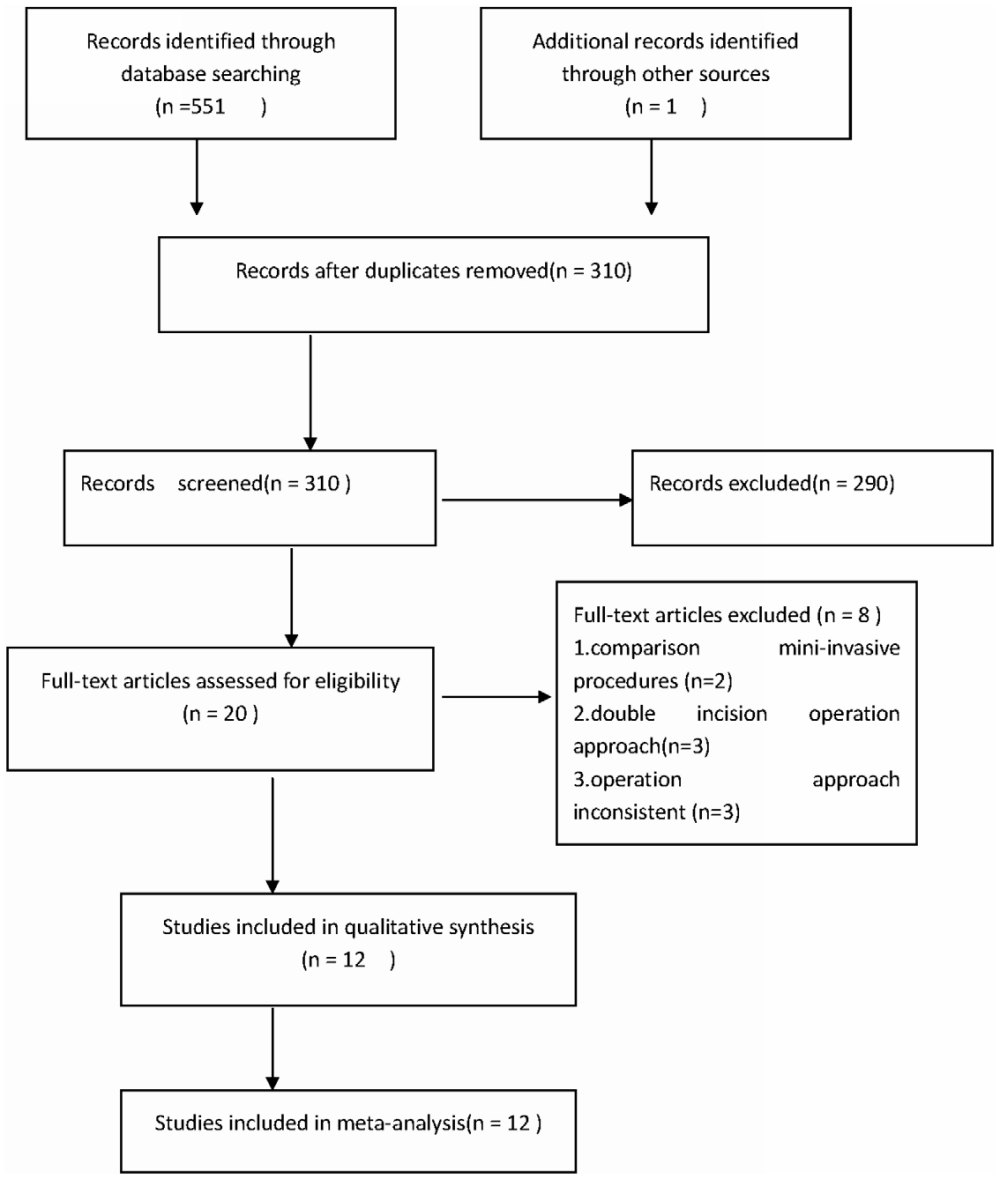
The process of identifying relevant studies is summarized.

A total of 552 citations were identified from the search strategy. Twelve studies were deemed appropriate (Figure 1). Eleven were written in English, one was in Chinese, and a total of 1077 cases were reported in the included studies. The basic characteristics of the 12 included studies are summarized in Table S2. The diseases included: osteoarthritis, rheumatoid arthritis, traumatic arthritis, congenital dislocation of the hip, femoral head necrosis, femoral neck fracture and other types. Consistent baselines were observed for all patients.

The surgical interventions included 4 studies that used the lateral approach [18], [19], [20], [21], 3 studies applied a posterior approach [22], [23], [24], 6 cases utilized the posterolateral approach [10], [11], [12], [18], [25], [26], and one case adopted the anterolateral approach [23]. mong them, the study by Goosen et al exercised both, the anterolateral approach and the posterior approach; Shitama et al also employed the posterolateral approach and the lateral approach; and 3 cases make use of the bone cement approach [10], [25], [23]. Follow-up studies of clinical outcomes and complications ranged between 6 weeks and 80 months.

### Risk of bias in included studies

**Figure 2:**
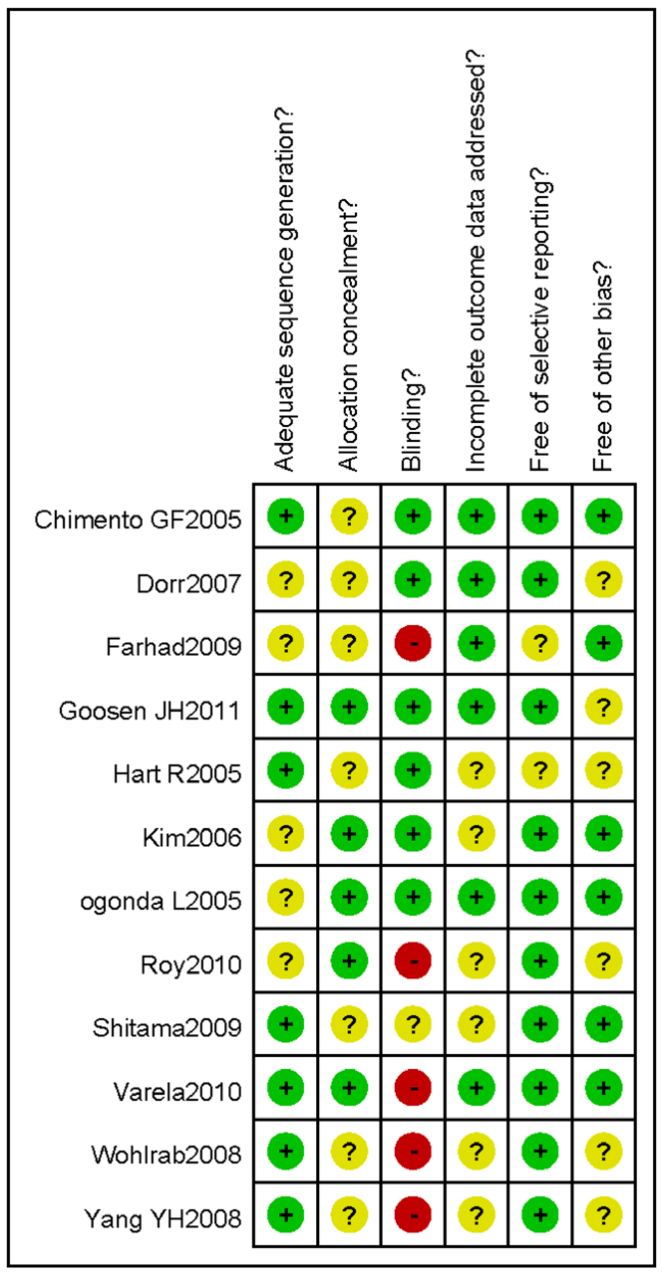
Risk of bias summary. A review of the authors' judgments about each risk of bias item for each included study. + is “yes”, − is “no”,?is “unclear”.

A summary of methodological domain assessment for each study is detailed in figure 2. Overall, the methodological quality of all the trials were found to be of medium risk of bias, The randomization technique was mentioned in 9 trials, which the randomized cohort picked a card, randomization number, or a randomization scheme. Six trials mentioned allocation concealment, 3 studies were single-blinded to the observers [10], [11], [25], and 2 studies were double-blinded to both the observers and the patients [22], [23]. All the studies could result in a potential selection bias. A variety of different THA prostheses were used in the studies reviewed which might result in a performance bias.

Although there were ‘lost to follow-up’ phenomenon in some studies, missing outcome data balanced in numbers across intervention groups, with similar reasons for missing data across groups, so attrition bias was considered as a low risk of bias.

### Publication bias analysis

**Figure 3:**
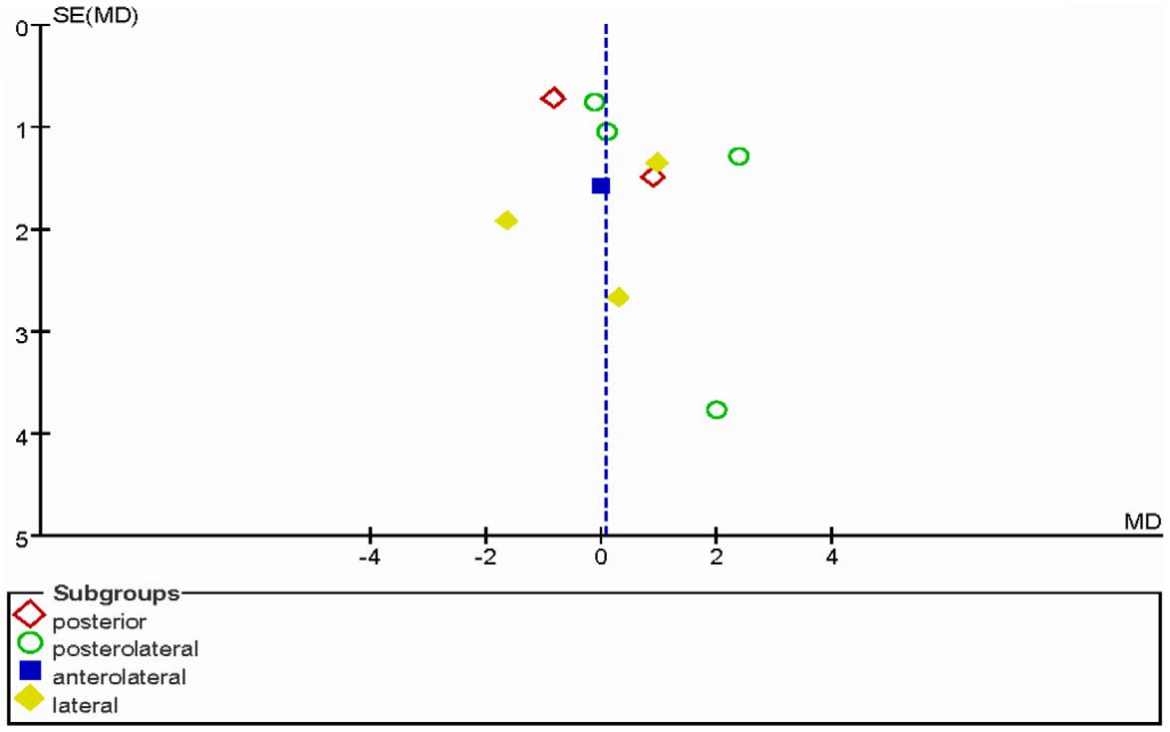
The funnel plot showing the publication bias of the subgroups of different surgical approaches for the most frequently reported outcome—acetabular cup abduction angle. SE (MD) standard error (mean difference).

Abduction angle of the acetabular cup was again used for the funnel plot analysis of publication bias (Figure 3), which revealed there was publication bias evident for the primary outcome frequency of the abduction angle.

### Primary outcomes: Imaging results

#### Posterolateral approach

**Figure 4:**
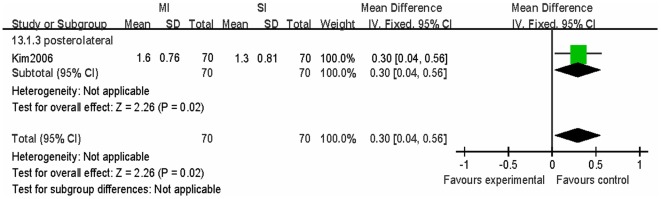
Forest plot of the differences in the increase of post-operative femoral offset in the MITHA and TTHA groups. The MITHA groups had significantly increased femoral offset than the TTHA groups (p = 0.02).

No significant differences were found between the MITHA and the TTHA groups in the imaging data of the acetabular cup abduction angle (p = 0.41), acetabular anteversion (p = 0.96), femoral prosthesis position (p = 0.83). The average femoral offset was significantly increased in the MITHA group than in the TTHA group (WMD = 3.00; 95% CI, 0.40–5.60; p = 0.02) (Tables S3, S4 and Figures 4).

No significant differences were noticed in the complications that occurred [dislocations (p = 0.24), nerve injury (p = 0.57), infection (p = 0.5), deep vein thrombosis (p = 1.00)], and revision rate (p = 0.44) (Tables S3 and S4).

#### Posterior approach

No significant differences were found between the MITHA and the TTHA groups in the imaging data of the acetabular cup abduction angle (p = 0.46), acetabular anteversion (p = 0.67), femoral prosthesis position (p = 0.25), and femoral offset (p = 0.16) (Tables S3 and S4).

No significant differences were found between the two groups in the complications that occurred [dislocations (p = 0.50), nerve injury (p = 0.50), infection (p = 0.46), deep vein thrombosis (p = 0.10), proximal femoral fracture (p = 0.73)], and revision rate (p = 0.41) (Tables S3 and S4).

#### Anterolateral approach

No significant differences were found between the two groups in the acetabular cup abduction angle (p = 1.00) and proximal femoral fracture (p = 0.16). However, data on acetabular anteversion, femoral prosthesis position, femoral offset, dislocations, nerve injury, infection, deep vein thrombosis, and revision rate were not adequate for analysis (Tables S3 and S4).

#### Lateral approach

No significant differences were found in the imaging data of the acetabular cup abduction angle (p = 0.88) and femoral prosthesis position (p = 0.30). Acetabular anteversion and femoral offset were not evaluated (Tables S3 and S4).

No significant differences were found among complications such as dislocations (p = 1.00), infection (p = 1.00), and deep vein thrombosis (p = 0.33). Nerve injury, proximal femoral fracture, and revision rate were not evaluated (Tables S3 and S4).

## Discussion

Minimally invasive total hip arthroplasty has often been the subject of debate in recent years. No clear definition exists for what constitutes MITHA, but there is a relative consensus that hip arthroplasties performed with any incision less than 10 centimeters can be included. Proponents of MITHA believe that this approach leads to a faster functional recovery, quicker hospital discharge, and increased patient satisfaction. Opponents believe that compared with TTHA, MITHA leads to increased iatrogenic nerve injury, prosthesis malposition, and revision rate, because of the limited field of vision during the surgery [6]–[8].

During THAs, the position of prosthesis is directly related to the efficacy of the procedure, and malposition of the prosthesis can cause dislocation, impaction, and pain post-operatively. The bony marks can be clearly visualized in TTHA. In contrast, any approach that is taken in MITHA, is plagued by limited surgical vision and inadequate exposure, thus resulting in difficult, and often, a malpositioning of the prosthesis. Hence, in our study, X-ray assessment of the acetabular abduction angle, anteversion, femoral prosthesis positions were generally considered reliable indicators of successful MITHA.

Total hip arthroplasty -related complications can severely compromise the efficacy of the surgery, such as infection, deep vein thrombosis, fracture adjacent to the prosthesis, and dislocation. The commonly seen complications after THA include dislocation (3.9%), pulmonary embolism (0.9%), and deep infection (0.2%) [27]. Woolson et al [7] described a retrospective cohort study of 135 hip arthroplasties and reported that their mini-incision group had a significantly higher risk of wound complications.

Minimally invasive surgeries for THAs can be divided into five categories: posterior, anterolateral (OCM: a modified Watson-Jones approach) [28], lateral and single anterior minimally invasive approach [29], two-incision muscle sparing approach [30] and posterior-lateral minimally invasive approach [31]. Surgical approaches can cause injuries to different soft tissues as they go through different tissue layers. In the past decade, the best surgical approach for THA has been controversially debated. Therefore, previous meta-analyses data of different approaches were not included in our analysis. In contrast, we divided the studies by the surgical approaches to gain a more objective result.

### Minimally invasive posterolateral approach

This approach can spare the gluteus medius and the hip flexors. In addition, this approach is a modification of the conventional posterolateral one and is easy for surgeons to master.

In this meta-analysis, no significant differences were found in the acetabular cup abduction angle (p = 0.41), acetabular anteversion (p = 0.96), and femoral prosthesis position (p = 0.83). In addition, there were no significant differences observed with respect to complications. However, one study found that the average femoral offset was significantly increased in the MITHA group (1.6±0.76 cm) than in the TTHA group (1.3±0.81 cm) (WMD = 3.00; 95% CI, 0.40–5.60; p = 0.02). Femoral offset is the distance from the center of rotation of the femoral head to a line bisecting the long axis of the femur. A decrease in the femoral offset moves the femur closer to the pelvis, which can result in impingemen at the extremes of movement. Moving the femur medially results in soft tissue laxity. Both of these problems can cause instability and possible dislocation [32], [33]. An increase in the femoral offset will move the femur laterally and will decrease impingement and improve soft tissue tension resulting in better stability without lengthening of the leg. However, excessive femoral offset can result in unevenly distributed stress, increased micromovement, thus increasing the risk of loose, osteolysis, and synovitis [34], [35]. This suggests a posterolateral minimally invasive approach may lead to better hip stability. At the same time, it must be pointed out, this result is only a research report with 140 patients and the results still need to be treated with caution.

### Minimally invasive posterior approach

In our meta-analysis, the results showed the radiological outcomes [acetabular cup abduction angle (p = 0.46), acetabular anteversion (p = 0.67), femoral prosthesis position (p = 0.25), femoral offset (p = 0.16)] and complications [dislocations (p = 0.50), nerve injury (p = 0.50), infection (p = 0.46), deep vein thrombosis (p = 0.10), proximal femoral fracture (p = 0.73), revision rate (p = 0.41)] were not statistically significant between the two treatments by the posterior approach. The possible reason might be that the posterior approach is well-established and the anatomical structures are well understood. The minimally invasive incision can easily be determined in THA through the posterior approach. Additionally, the correct use of retractors can increase the operation field. Particularly, this approach can clearly expose the medullary cavity of the femur at the proximal end of the incision, and expose the acetabulum side at the distal end of the incision. The long-term effects, however, still need further observation.

### Minimally invasive anterolateral and lateral approach

The minimally invasive anterolateral and lateral approach was thought to be difficult to perform as the acetabular cup installation was prone to excessive anteversion and abduction, causing post-operative anterior dislocation [36]. Siguier et al. summarized 1037 cases of total hip replacements performed by the anterolateral approach and concluded that this procedure can best spare the external rotators of the hip, fascia lata, great trochanter, and gluteal muscles, thus minimizing the damage to soft tissues adjacent to the hip and maintaining the balance in soft tissues, without increasing the risk of dislocation.

In our meta-analysis, only the acetabular cup abduction angle (p = 1.00) and proximal femoral fracture (p = 0.16) were analyzed and no statistical significance was found (p>0.05). However, no adequate data was available to effectively evaluate the acetabular anteversion, femoral prosthesis position, femoral offset, dislocations, nerve injury, infection, deep vein thrombosis, and revisions. Therefore, no positive conclusion can be drawn from the current study until more high-quality randomized controlled studies are available.

The lateral approach also has a relatively lower risk of postoperative dislocation. he acetabulum can be adequately exposed and the prosthesis can be easily implanted. Nevertheless, the major shortcoming of this procedure is that part of the gluteus medius and gluteus minimus is spliced, causing weakness of the abductor, damage to the superior gluteal nerve, ipsilateral post-operative lameness, and increased risk of heterotopic ossification around the hip [37]. In addition, this procedure has poor exposure when manipulating the prosthesis and the retractor often causes severe damage to the skin. In this study, acetabular anteversion, femoral offset, nerve injury, proximal femoral fracture, and revision were not evaluated and therefore the safety profile is not confirmed yet.

Our findings are mainly limited by the quality and the low number of included studies. This limited our assessment of potential publication bias and unpublished research having negative results that cannot be identified. Therefore, publication bias may exist, which could result in the overestimation of the effectiveness of interventions. Third, the methodological quality of the all trials was found to be medium risk of bias. Due to these limitations, the combined results of this meta-analysis should be cautiously accepted, and more independent high-quality RCTs with effectiveness analyses are needed.

### Conclusion

Our meta-analysis indicates that the posterior approach in MITHA is a safe surgical procedure, without increased operative complication rates and component malposition rates. The posterolateral approach may lead to increased femoral offset. No thorough conclusion can be drawn from the lateral or anterolateral approach as the risks of adverse effects and complications are increased at the site of prosthesis. It must be pointed out, that this result is only a research report from140 patients and the results need to be treated with caution. More high-quality studies are needed to assess the best surgical approach in minimally invasive hip arthroplasties.

## Supporting Information

Table S1 The Cochrane Collaboration's tool for assessing risk of bias.
(DOC)

Table S2 Characteristics of included randomized controlled trials (RCTs).
(DOC)

Table S3 Minimally invasive versus traditional total hip arthroplasty (radiological outcomes).
(DOC)

Table S4 Minimally invasive versus traditional total hip arthroplasty (complications outcomes).
(DOC)

## References

[1] Berry DJ, Berger RA, Callaghan JJ, Dorr LD, Duwelius PJ (2003). Minimally invasive total hip arthroplasty. Development, early results, and a critical analysis.. J Bone Joint Surg Am.

[2] Sculco TP, Jordan LC, Walter WL (2004). Minimally invasive total hip arthroplasty : the Hospital for Special Surgery experience.. Orthop Clin North Am.

[3] Szendroi M, Sztrinkai G, Vass R, Kiss J (2006). The impact of minimally invasive total hip arthroplasty on the standard procedure.. Int Orthop.

[4] Wall SJ, Mears SC (2008). Analysis of published evidence on minimally invasive total hip arthroplasty.. J Arthroplasty.

[5] Pagnano MW, Hebl J, Horlocker T (2006). Assuring a painless total hip arthroplasty: a multimodal approach emphasizing peripheral nerve blocks.. J Arthroplasty.

[6] Nakamura S, Matsuda K, Arai N, Wakimoto N, Matsushita T (2004). Mini-incision posterior approach for total hip arthroplasty.. Int Orthop.

[7] Woolson ST, Mow CS, Syquia JF, Lannin JV, Schurman DJ (2004). Comparison of primary total hips replacements performed with standard incision or mini-incision.. J Bone Joint Surg.

[8] Bradley P, Graw MD, Steven T, Woolson MD, Heather G (2010). Minimal Incision Surgery as a Risk Factor for Early Failure of Total Hip Arthroplasty.. Clin Orthop Relat Res.

[9] Bennett D, Ogonda L, Elliot D, Humphreys L, Lawlor M (2007). Comparison of immediate postoperative walking ability in patients receiving minimally invasive and standard-incision hip arthroplasty: a prospective blinded study.. J Arthroplasty.

[10] Chimento GF, Pavone V, Sharrock N, Kahn B, Cahill J (2005). Minimally invasive total hip arthroplasty: a prospective randomized study.. J Arthroplasty.

[11] Kim YH (2006). Comparison of primary total hip arthroplasties performed with a minimally invasive technique or a standard technique: a prospective and randomized study.. J Arthroplasty.

[12] Ogonda L, Wilson R, Archbold P, Lawlor M, Humphreys P (2005). A minimal-incision technique in total hip arthroplasty does not improve early postoperative outcomes: a prospective, randomized, controlled trial.. J Bone Joint Surg Am.

[13] Cheng T, Feng JG, Liu T, Zhang XL (2009). Minimally invasive total hip arthroplasty: a systematic review.. Int Orthop.

[14] Smith TO, Blake V, Hing CB (2011). Minimally invasive versus conventional exposure for total hip arthroplasty: a systematic review and meta-analysis of clinical and radiological outcomes.. Int Orthop.

[15] Khan RJ (2011). Comments on Smith et al: Comments on Smith et al.: minimally invasive versus conventional exposure for total hip arthroplasty: a systematic review and meta-analysis of clinical and radiological outcomes.. Int Orthop.

[16] Higgins J, Green S (2008). Cochrane Handbook for Systematic Reviews of Interventions Version 5.0.0.. http://www.cochrane-handbook.org.

[17] Moher D, Cook DJ, Eastwood S, Olkin I, Rennie D (1999). Improving the quality of reports of meta-analyses of randomized controlled trials: the QUOROM statement. Quality of reporting of meta-analyses.. Lancet.

[18] Shitama T, Kiyama T, Naito M, Shiramizu K, Huang G (2009). Which is more invasive-mini versus standard incisions in total hip arthroplasty?. Int Orthop.

[19] Varela-Egocheaga J R, Suárez-Suárez M A, Fernandez-Villan M (2010). Minimally invasive lateral approach in total hip replacement: a prospective randomised study.. Rev Esp Cir Ortop Traumatol.

[20] Mazoochian F, Weber P, Schramm S, Utzschneider S, Fottner A (2009). Minimally invasive total hip arthroplasty: a randomized controlled prospective trial.. Arch Orthop Trauma Surg.

[21] Wohlrab D, Droege JW, Mendel T, Brehme K, Riedl K (2008). Minimally invasive vs. transgluteal total hip replacement. A 3-month follow-up of a prospective randomized clinical study.. Orthopäde.

[22] Dorr LD, Maheshwari AV, Long WT, Wan Z, Sirianni LE (2007). Early pain relief and function after posterior minimally invasive and conventional total hip arthroplasty. A prospective, randomized, blinded study.. J Bone Joint Surg Am.

[23] Goosen JH, Kollen BJ, Castelein RM, Kuipers BM, Verheyen CC (2011). Minimally invasive versus classic procedures in total hiparthroplasty: a double-blind randomized controlled trial.. Clin Orthop Relat Res.

[24] Roy L, Laflamme GY, Carrier M, Kim PR, Leduc S (2010). A randomised clinical trial comparingminimally invasive surgery to conventional approach for endoprosthesis in elderly patients with hip fractures.. Injury.

[25] Hart R, Stipcák V, Janecek M, Visna P (2005). Component position following total hip arthroplasty through a miniinvasive posterolateral approach.. Acta Orthop Belg.

[26] Yang YH, Gao ZL, Wang JC (2008). Mini—incision Total Hip Replacement for Femoral Neck Fracture in Senile Patients.. Chinese Journal of bone and joint injury.

[27] Phillips CB, Barrett JA, Losina E, Mahomed NN, Lingard EA (2003). Incidence rates of dislocation, pulmonary embolism, and deep infection during the first six months after elective total hip replacement.. J Bone Joint Surg (Am).

[28] Bertin KC, Röttinger H (2004). Anterolateral mini-incision hip replacement surgery: a modified Watson-Jones approach.. Clin Orthop Relat Res.

[29] Siguier T, Siguier M, Brumpt B (2004). Mini-incision anterior approach does not increase dislocation rate: a study of 1037 total hip replacements.. Clin Orthop Relat Res.

[30] Berger RA (2004). The technique of minimally invasive total hip arthroplasty using the two-incision approach.. Instr Course Lect.

[31] Goldstein WM, Branson JJ (2004). Posterior-lateral approach to minimal incision total hip arthroplasty.. Orthop Clin North Am.

[32] Morrey BF (1997). Dificult complications after hip replacem.. Clin Orthop.

[33] Dorr LD, Wan Z (1998). Causes of and treatment protocol for instability of total hip replacement.. Clin Orthop Relat Res.

[34] Cannestra VP, Berger RA, Quigley LR, Jacobs JJ, Rosenberg AG (2000). Total hip arthroplasty with a precoated ofset stem. Four to nine—year results.. J Bone Joint Surg Am.

[35] Kleemann RU, Heller MO, Stoeckle U, Taylor WR, Duda GN (2003). THA loading arising from increased femoral an terversion and ofset may lead to critical cement stresses.. Onhop Res.

[36] Yang CF, Zhang DW, Niu S, Kong LZ, Zhu JY (2009). Curative Effect of Minimally-invasive Total Hip Arthroplasty.. Chinese Journal of bone and joint injury.

[37] Archibeck MJ, Berger RA, Jacobs JJ, Quigley LR, Gitelis S (2001). Second- generation cementless total.hip arthroplasty.. Eight to eleven- year results.

