# Supplementary material for: Minimally Invasive Surgical Approaches and Traditional Total Hip Arthroplasty: A Meta-Analysis of Radiological and Complications Outcomes
Source: PLoS One. 2012 May 24;7(5):e37947. doi: 10.1371/journal.pone.0037947 (PMC3360020; doi:10.1371/journal.pone.0037947)
Supplement: Table S1 — The Cochrane Collaboration's tool for assessing risk of bias. (DOC) [file pone.0037947.s001.doc]

Table 1 The Cochrane Collaboration’s tool for assessing risk of bias

| **Domain** | **Description** | **Review authors’ judgement** |
| --- | --- | --- |
| **Sequence generation** | Describe the method used to generate the allocation sequence in sufficient detail to allow an assessment of whether it should produce comparable groups. | Was the allocation sequence adequately generated?  (Yes/No/Unclear) |
| **Allocation concealment.** | Describe the method used to conceal the allocation sequence in sufficient detail to determine whether intervention allocations could have been foreseen in advance of, or during, enrolment | Was allocation adequately concealed?  (Yes/No/Unclear) |
| **Blinding of participants,**  **personnel and outcome** | Describe all measures used, if any, to blind study participants and personnel from knowledge of which intervention a participant received. Provide any information relating to whether the intended blinding was effective. | Was knowledge of the allocated intervention  adequately prevented during the study?  (Yes/No/Unclear) |
| **Incomplete outcome data** | Describe the completeness of outcome data for each main outcome, including attrition and exclusions from the analysis. State whether attrition and exclusions were reported, the numbers in each intervention group (compared with total randomized participants), reasons for attrition/exclusions where reported, and any re-inclusions in analyses performed by the review authors. | Were incomplete outcome data adequately addressed?  (Yes/No/Unclear) |
| **Selective outcome reporting.** | State how the possibility of selective outcome reporting was examined by the review authors, and what was found. | Are reports of the study free of suggestion of selective outcomereporting?  (Yes/No/Unclear) |
