# Supplementary material for: Minimally Invasive Surgical Approaches and Traditional Total Hip Arthroplasty: A Meta-Analysis of Radiological and Complications Outcomes
Source: PLoS One. 2012 May 24;7(5):e37947. doi: 10.1371/journal.pone.0037947 (PMC3360020; doi:10.1371/journal.pone.0037947)
Supplement: Table S2 — Characteristics of included randomized controlled trials (RCTs). (DOC) [file pone.0037947.s002.doc]

Table 2 .Characteristics of included randomized controlled trials (RCTs)

| **Study** | Methods | Participants | Interventions | **Outcome** |
| --- | --- | --- | --- | --- |
| **Chimento**  **et al** | RCT;  FU=2 years | OA=28MI vs 32 SI | MI posterolateral 8cm vs SI posterolateral 15cm;press-fit cup cemented or press-fit stem | acetabular cup abduction angle, femoral prosthesis position, dislocation, nerve injury, revision. |
| **Dorr**  **et al** | RCT;  FU=3months | OA,PA,HD,ON=30MI vs 30 SI; | MI posterior 10±2 cm vs SI posterior20±2cm;cementless cup noncemented stem | acetabular cup abduction angle, acetabular anteversion, infection, deep vein thrombosis, Proximal femoral fracture |
| **Farhad**  **et al** | RCT;  FU=3months | 26 MI vs 26 SI | MI lateral 8.9cm vs SI lateral 14cm, cementless THA | acetabular cup abduction angle, femoral prosthesis position, dislocation, infection, deep vein thrombosis |
| **Goosen**  **et al** | RCT；  FU=1 year | 60 MI vs 60 SI | MI anterolateral/posterior vs SI anterolateral/posterior cementless THA | acetabular cup abduction angle, nerve injury, infection, deep vein thrombosis, Proximal femoral fracture, revision. |
| **Hart et al** | RCT;  FU=39months | OA=60 MI vs 60 SI; | MI posterolateral 9–10cm vs SI posterolateral 20cm; cemented THA ; | acetabular cup abduction angle,, acetabular anteversion, femoral prosthesis position, dislocation, infection |
| **Kim et al** | RCT;  FU=26.4months | ON, OA, AS=70 MI vs 70 SI | MI posterolateral 8.8±1.5 cm vs SI posterolateral 23.0±2.1cm cementless cup and cementless stem; | acetabular cup abduction angle, femoral prosthesis position, The averagefemoral offset was increased, dislocation, nerve injury, infection |
| **Ogonda et al** | RCT;  FU= 6 weeks | OA , ON ,RA=109 MI vs 110 SI; | MI posterolateral 9.5±0.95 cm vs SI posterolateral 15.81±0.93 cm cementless cup cemented stem; | acetabular cup abduction angle, femoral prosthesis position, dislocation |
| **Roy et al** | RCT;  FU=2 years | FNF= 25 MI vs 31SI | MI posterior 8cm vs SI posterior≥16cm;Cemented THA | femoral prosthesis position, dislocation |
| **Shitama et al** | RCT;  FU= 6 months | 34 MI vs 28 SI | MI posterolateral/ translateral vs SIposterolateral/ translateral cementless THA | Infection, Proximal femoral fracture |
| **Varela et al** | RCT;  FU=12 months | OA=25 MI vs 25 SI | MI lateral ≤10cm vs SI lateral non-cemented femoral component | acetabular cup abduction angle, femoral prosthesis position, infection |
| **Wohlrab et al** | RCT;  FU=3 months | OA=20MI vs 20SI | MI lateral vs SI lateral cementless THA | Acetabular cup abduction angle, acetabular anteversion |
| **Yang et al** | RCT;  FU= 80months | FNF= 30 MI vs 30 SI | MI posterolateral 8.52±1.62cm vs SI posterolateral 18.5±3.12 cm Cemented THA | deep vein thrombosis |

MI mini incision, SI standard incision, OA osteoarthritis, RA rheumatoid arthritis, PA posttraumatic arthritis, HD hip dysplasia, ON osteonecrosis, FNF femoral neck fracture, FU follow-up
