# Supplementary material for: Minimally Invasive Surgical Approaches and Traditional Total Hip Arthroplasty: A Meta-Analysis of Radiological and Complications Outcomes
Source: PLoS One. 2012 May 24;7(5):e37947. doi: 10.1371/journal.pone.0037947 (PMC3360020; doi:10.1371/journal.pone.0037947)
Supplement: Table S3 — Minimally invasive versus traditional total hip arthroplasty (radiological outcomes). (DOC) [file pone.0037947.s003.doc]

Table 3 Minimally invasive versus traditional total hip arthroplasty (radiological outcomes)

| Outcome or subgroup title | No. of  studies | No. of  participans | OR/WMD(95%CI) | p-value |
| --- | --- | --- | --- | --- |
| **1**.acetabular cup abduction angle  1.1 posterior approach  1.2 posterolateral approach  1.3 anterolateral approach  1.4 lateral approach | 2  4  1  3 | 279  334  94  142 | -0.48 [-1.75, 0.79]  0.46 [-0.63, 1.54]  0.00 [-3.09, 3.09]  0.16 [-1.85, 2.16] | 0.46  0.41  1.00  0.88 |
| **2**. acetabular anteversion  2.1 posterior approach  2.2 posterolateral approach  2.3 anterolateral approach  2.4 lateral approach | 1  2  0  0 | 60  160  0  0 | 0.50 [-1.81,2.81]  0.04 [-1.55,1.63]  Not estimable  Not estimable | 0.67  0.96  Not estimable  Not estimable |
| **3**.femoral prosthesis position  3.1posterior approach  3.2 posterolateral approach  3.3 anterolateral approach  3.4 lateral approach | 2  3  0  2 | 270  252  0  102 | 0.59 [0.24, 1.45]  0.89 [0.33, 2.42]  Not estimable  0.58 [0.20, 1.64] | 0.25  0.83  Not estimable  0.30 |
| **4**. Femoral offset  4.1 posterior approach  4.2 posterolateral approach  4.3 anterolateral approach  4.4 lateral approach | 1  1  0  0 | 60  140  0  0 | 2.20 [-0.85, 5.25]  3.00 [0.40, 5.60]  Not estimable  Not estimable | 0.16  0.02  Not estimable  Not estimable |

NO number, OR odds ratio, WMD weighed mean difference
