# Supplementary material for: Minimally Invasive Surgical Approaches and Traditional Total Hip Arthroplasty: A Meta-Analysis of Radiological and Complications Outcomes
Source: PLoS One. 2012 May 24;7(5):e37947. doi: 10.1371/journal.pone.0037947 (PMC3360020; doi:10.1371/journal.pone.0037947)
Supplement: Table S4 — Minimally invasive versus traditional total hip arthroplasty (complications outcomes). (DOC) [file pone.0037947.s004.doc]

Table 4. Minimally invasive versus traditional total hip arthroplasty (complications outcomes)

| Outcome or subgroup title | No. of  studies | No. of  participans | OR/WMD(95%CI) | p-value |
| --- | --- | --- | --- | --- |
| **1**. dislocations  1.1 posterior  1.2 posterolateral  1.3 anterolateral  1.4 lateral | 2  4  0  1 | 275  382  0  52 | 0.33 [0.01, 8.12]  2.68 [0.52, 13.91]  Not estimable  1.00 [0.06, 16.89] | 0.50  0.24  Not estimable  1.00 |
| **2**. nerve injury  2.1 posterior  2.2 posterolateral  2.3 anterolateral  2.4 lateral | 1  2  0  0 | 120  200  0  0 | 3.05 [0.12, 76.39]  1.79 [0.23, 13.68]  Not estimable  Not estimable | 0.50  0.57  Not estimable  Not estimable |
| **3**. infection  3.1 posterior  3.2 posterolateral  3.3 anterolateral  3.4 lateral | 4  3  0  2 | 455  322  0  102 | 0.55 [0.12, 2.63]  3.04 [0.12, 75.99]  Not estimable  3.12 [0.31, 31.00] | 0.46  0.5  Not estimable  0.33 |
| **4**. deep vein thrombosis  4.1 posterior  4.2 posterolateral  4.3 anterolateral  4.4 lateral | 2  1  0  1 | 180  60  0  52 | 1.00 [0.14, 7.23]  1.00 [0.06, 16.76]  Not estimable  3.12 [0.12, 80.12] | 1.00  1.00  Not estimable  0.69 |
| **5**. Proximal femoral fracture  5.1 posterior  5.2 posterolateral  5.3 anterolateral  5.4 lateral | 2  0  1  0 | 180  0  62  0 | 1.26 [0.33, 4.85]  Not estimable  8.41 [0.43, 163.27]  Not estimable | 0.73  Not estimable  0.16  Not estimable |
| **6**. revision  6.1 posterior  6.2 posterolateral  6.3 anterolateral  6.4 lateral | 1  1  0  0 | 120  60  0  0 | 2.07 [0.36, 11.76]  3.55 [0.14, 90.59]  Not estimable  Not estimable | 0.41  0.44  Not estimable  Not estimable |

NO number, OR odds ratio, WMD weighed mean difference
